# Impact of TOF on Brain PET With Short-Lived ^11^C-Labeled Tracers Among Suspected Patients With AD/PD: Using Hybrid PET/MRI

**DOI:** 10.3389/fmed.2022.823292

**Published:** 2022-03-02

**Authors:** D.D.N Wimalarathne, Weiwei Ruan, Xun Sun, Fang Liu, Yongkang Gai, Qingyao Liu, Fan Hu, Xiaoli Lan

**Affiliations:** ^1^Department of Nuclear Medicine, Union Hospital, Tongji Medical College, Huazhong University of Science and Technology, Wuhan, China; ^2^Hubei Province Key Laboratory of Molecular Imaging, Union Hospital, Tongji Medical College, Huazhong University of Science and Technology, Wuhan, China; ^3^Department of Radiography and Radiotherapy, Faculty of Allied Health Sciences, General Sir John Kotelawala Defence University, Rathmalana, Sri Lanka

**Keywords:** time-of-flight, PET/MRI, quantification, SUV, reconstruction, ^11^C-labeled tracers

## Abstract

**Objective:**

To explore the impact of the time-of-flight (TOF) reconstruction on brain PET with short-lived ^11^C-labeled tracers in PET magnetic resonance (PET/MR) brain images among suspected patients with Alzheimer's and Parkinson's disease (AD/PD).

**Methods:**

Patients who underwent ^11^C-2-ß-carbomethoxy-3-b-(4-fluorophenyl) tropane (^11^C-CFT) and 2-(4-N-[^11^C] methylaminophenyl)-6-hydroxybenzothiazole (^11^C-PiB) PET/MRI were retrospectively included in the study. Each PET LIST mode data were reconstructed with and without the TOF reconstruction algorithm. Standard uptake values (SUVs) of Caudate Nucleus (CN), Putamen (PU), and Whole-brain (WB) were measured. TOF and non-TOF SUVs were assessed by using paired *t*-test. Standard formulas were applied to measure contrast, signal-to-noise ratio (SNR), and percentage relative average difference of SUVs (%RAD-SUVs).

**Results:**

Total 75 patients were included with the median age (years) and body mass index (BMI-kg/m^2^) of 60.2 ± 10.9 years and 23.9 ± 3.7 kg/m^2^ in ^11^C-CFT (*n* = 41) and 62.2 ± 6.8 years and 24.7 ± 2.9 kg/m^2^ in ^11^C-PiB (*n* = 34), respectively. Higher average SUVs and positive %RAD-SUVs were observed in CN and PU in TOF compared with non-TOF reconstructions for the two ^11^C-labeled radiotracers. Differences of SUV_mean_ were significant (*p* < 0.05) in CN and PU for both ^11^C-labeled radiotracers. SUV_max_ was enhanced significantly in CN and PU for ^11^C-CFT and CN for ^11^C-PiB, but not in PU. Significant contrast enhancement was observed in PU for both ^11^C-labeled radiotracers, whereas SNR gain was significant in PU, only for ^11^C-PiB in TOF reconstruction.

**Conclusion:**

Time-of-flight leads to a better signal vs. noise trade-off than non-TOF in ^11^C-labeled tracers between CN and PU, improving the SUVs, contrast, and SNR, which were valuable for reducing injected radiation dose. Improved timing resolution aided the rapid decay rate of short-lived ^11^C-labeled tracers, and it shortened the scan time, increasing the patient comfort, and reducing the motion artifact among patients with AD/PD. However, one should adopt the combined TOF algorithm with caution for the quantitative analysis because it has different effects on the SUV_max_, contrast, and SNR of different brain regions.

## Introduction

Alzheimer's disease (AD) and Parkinson's disease (PD) are the most common neurodegenerative diseases in the elderly. AD is caused due to abnormal build-up of amyloid and tau proteins in and around the neurons which disrupt the function of the neurons by triggering the neuronal damage or eventually dead cells, particularly in the cortex and hippocampus, whereas PD affects predominantly dopaminergic neurons in a specific area in the brain called substantia nigra (1, 2). In the diagnosis of AD/PD or differentiation of mild cognitive impairment (MCI) and AD from normal aging, it is essential to investigate the variations of metabolic activity and characteristic patterns of radiotracers with PET in key brain regions (3–7).

In recent years, the hybrid PET/MRI was developed successfully and has entered clinical practice. The hybrid PET/MRI, like two high-end technologies, can simultaneously obtain images of PET and MRI, which provide excellent anatomic information and functional MRI parameters with the metabolic and molecular information as a one-stop-shop. Therefore, the hybrid PET/MRI has always been used for neurodegenerative diseases, especially showing great potential for differential diagnosis of early AD/PD with some specific PET tracer (8–10). Among a tracer targeted dopamine transporters (DATs) level for early diagnosis of PD is ^11^C labeled cocaine derivative, i.e., ^11^C-2-ß-carbomethoxy-3-b-(4-fluorophenyl) tropane (^11^C-CFT) (8). In the patients with PD, the DATs level will change, and the PET images with ^11^C-CFT will show the asymmetrical reduction in the caudate nucleus (CN) or putamen (PU). The Pittsburgh compound B, i.e., 2-(4-N-[^11^C] methylaminophenyl)-6-hydroxybenzothiazole (^11^C-PiB) is a benzothiazole derivative of thioflavin T that is used to image beta-amyloid deposits in AD (9), and the corresponding PET images with ^11^C-PiB will show the diffuse uptake in the brain. However, the synthesis and quality control of ^11^C-CFT and ^11^C-PiB are complicated processes which are followed by high-performance liquid chromatography (HPLC) purification (9, 10). As well, ^11^C radioisotope tends to decay fast within a half-life (**T**_**1/2**_) of 20.38 min. In theory **T**_**1/2**_ **= 0.693/λ** where **λ** is the decaying constant and radioactivity (**A**) at a “**t**” time measured by **A = A**_**0**_**e**^**λt**^ where **A**_**0**_is the radioactivity at time zero (**t = 0**). Accordingly, shorten T_1/2_ tends to decrease the **A** at a given time elapsed. Hence, improvising the PET image acquisition and reconstruction became more important for brain PET with short-lived radiotracers. Uptake time of said ^11^C-labeled tracers is ~40–60 min as mentioned in Table 1, where after consecutive 2T_1/2_ to 3T_1/2_ it remains A_0_/4 to A_0_/8 of original radioactivity within the body. Thus, it is found challenging to image under a low count field with existing conventional PET scanners (11).

**Table 1:** Basic information of the patient.

**Patient Information**	**^**11**^C-CFT**	**^**11**^C-PiB**
Patients included (n)	41	34
Age (y)	60.2 ± 10.9	62.2 ± 6.8
BMI (kg/m^2^)	23.9 ± 3.7	24.7 ± 2.9
Injected Dose/Weight (MBq/kg)	3.9 ± 1.4	4.3 ± 1.1
Mean uptake time (min)	54.4 ± 15.9	43.8 ± 19.5

Clinical PET image quality has drastically improved by utilizing several advanced reconstruction techniques, i.e., time-of-flight (TOF) reconstruction technology (12). The TOF system measures and records the time difference of two coincident photons and improves the activity localization by more accurately identifying an annihilation event along a line of response (LOR) (13). Thus, TOF effects on the gain in signal-to-noise ratio (SNR) (14) further, TOF results in a faster and more uniform convergence with three-dimensional (3D) iterative reconstruction (15).

In the past decade, it was proved that larger patients (BMI ≥ 25.0 kg/m^2^) are benefitted from the TOF technique (16). Since, TOF reconstruction acts as a weight equalizer, gaining consistent image quality among patients, regardless of weight and size (17). Improved small lesion detection is reported among several TOF PET/MRI studies (18–22). Further improved TOF contributes in the reduction of injected radiation dose to the patient, so as lowering the radiation dose to the medical and general public (23). Budinger et al. elaborated that the TOF sensitivity gain equal to **D/Δx** (**D** is the object diameter and Δx =(C^*^Δt/2) where **C** is the speed of the light and **Δt** is the full-width at half-maximum (FWHM) of the timing resolution of the scanner (24). Accordingly, it is proven that TOF gain is inversely proportionate with the time resolution of the PET detector system. The time resolution was significantly improved by the invention of newer embedded semiconductor detectors (e.g., SiPM) for PET by featuring TOF in PET/MR systems (15, 25, 26). Since scan time is reduced while keeping the same image quality (11). Subsequently, TOF vs. brain PET clinical studies were conducted in recent years (27, 28). Yet, to our knowledge, few studies have been conducted to assess the TOF reconstruction techniques for brain PET with short-lived ^11^C-labeled tracers (29).

This study explored the effects of the TOF reconstruction technique on brain PET quantification with short-lived ^11^C-CFT and ^11^C-PiB in hybrid PET/MR brain imaging among suspected patients with AD/PD. Since ^11^C-PiB showed a diffuse uptake throughout the whole-brain, the quantification evaluation for PET with TOF reconstruction was carried for the whole brain. The ^11^C-CFT uptake was mainly focused on CN and PU regions. Hence, we evaluated the effect of TOF reconstruction on CN and PU volume of interests (VOIs) for ^11^C-CFT brain PET, and the effects on CN and PU VOIs for ^11^C-PiB brain PET were also further evaluated for comparing with the results from ^11^C-CFT to investigate whether the effects of TOF technique were related with different tracer. The key purpose of this study was to determine if quantification differences are present in TOF compared with the non-TOF technique among short-lived ^11^C- labeled radiopharmaceuticals in PET/MRI brain imaging.

## Methods

### Ethical Statement

This retrospective experimental study of exploring the effects of the TOF reconstruction technique on brain PET quantification with ^11^C-CFT and ^11^C-PiB in hybrid PET/MR brain imaging among suspected patients with AD/PD performed at our institute, which has been approved by the Institutional Review Board of Union Hospital, Tongji Medical College, Huazhong University of Science and Technology. The need for written informed consent was waived.

### Subjects

Patients' studies of suspected AD/PD referred for ^11^C-CFT and ^11^C-PiB PET/MRI (*n* = 75) were retrieved by an independent data analyst prior to automated standard uptake value (SUV) analysis by using the PNEURO module of PMOD 3.906 software. The corresponding detailed information for the subjects is shown in Table 1.

### PET/MRI

All acquisitions of patients were performed on a SIGNA TOF-PET/MRI (GE Healthcare, Waukesha, WI, USA) with subsequent specifications: 130 cm × 60 cm × 60 cm bore dimension, 3.0 Tesla superconductive magnet, gradient coils: 44 mT/m peak amplitude, and 200 T/m/s peak slew rate, Detector type: SiPM, TOF (timing resolution for fast TOF performance <400 ps), Cryogen Type: Liquid Helium. The mean injected radiation dose (MBq/kg) and uptake time (minutes) for the two radiotracers are mentioned in Table 1. All patients were asked to void before scanning began. Prior to PET/MRI, patients were given an instruction sheet and an informed consent form to fill and to be submitted. Claustrophobic patients, patients with metal implants, and uncooperative patients were excluded from the investigations. The PET/MR 8-channel brain coil with a mirror was placed on the table on top of the adaptor. Patients were instructed verbally to keep the body aligned 90 degrees to the midsagittal plane in the supine position, hands alongside the trunk, and stay still 10 and 20 min for ^11^C-CFT and ^11^C-PiB, respectively. MRI was performed with T1-weighted imaging (3D gradient-echo sequence, flip angle = 12 degrees, time of echo [TE]/time of repetition [TR] = 2.6/6.9 ms, bandwidth = 50 KHz, field of view (FOV) = 24 cm × 24 cm, matrix = 384 × 384) during the ^11^C-CFT and ^11^C-PiB PET scanning.

### PET Reconstruction

The PET images were reconstructed by using the ordered subsets expectation maximum (OSEM) algorithm with the TOF technique and non-TOF technique, respectively. The other parameters were same as followed: FOV = 30 cm × 30 cm, matrix = 192 × 192, filter cutoff = 3.0 mm, subsets = 28, iterations = 3. Gaussian post-reconstruction filtering with a 3.0 mm full width of half maximum (FWHM) was used to improve the image SNR. In all cases, the PET attenuation correction was atlas-based MRI attenuation correction, combined with Dixon water-fat separation methods (30). The additional corrections to scatter, random events, and dead-time were applied accordingly.

### Image Analysis Semi-Quantitative Analysis

**Figure 1:**
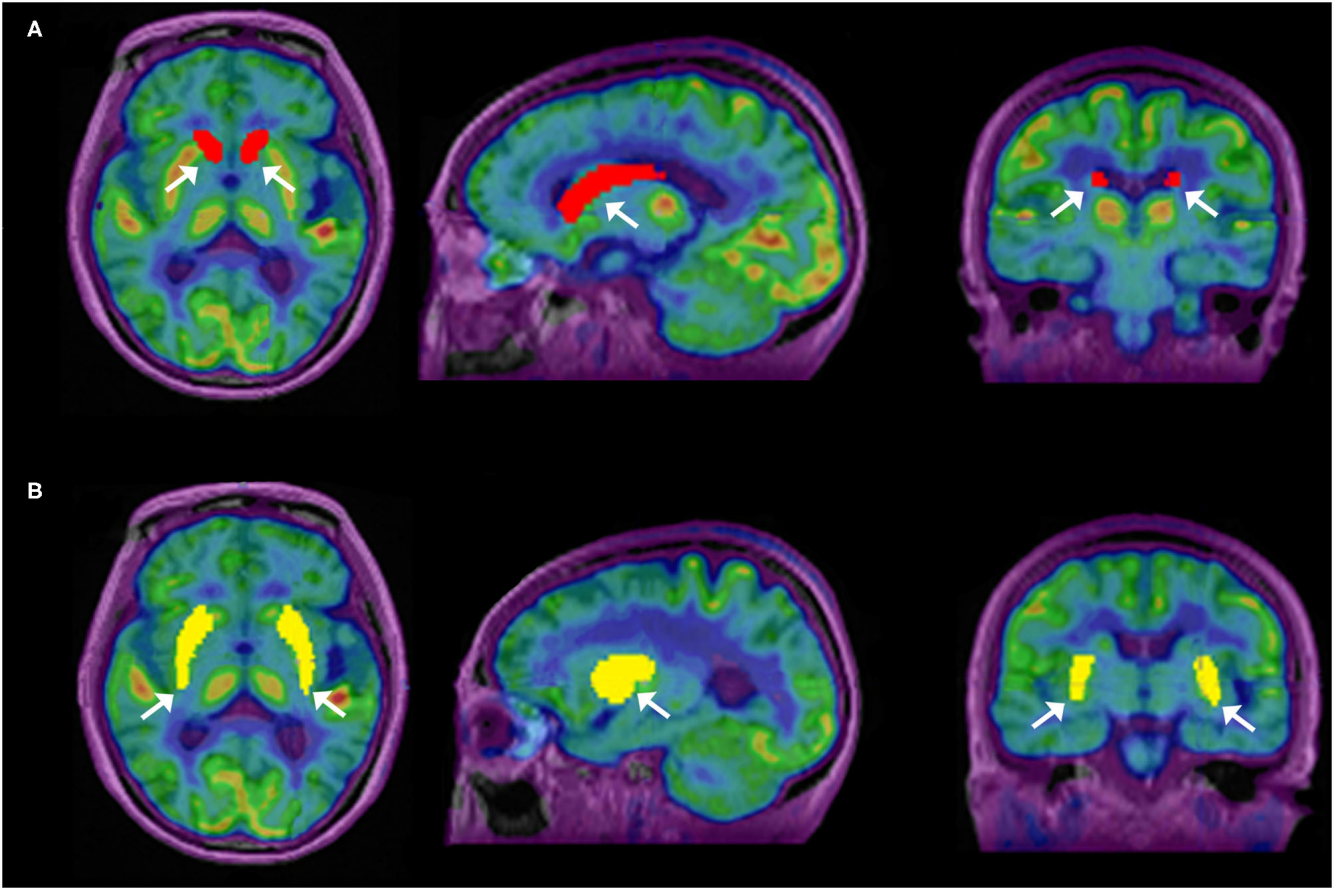
Segmentation of caudate nuclei (CN) and putamen (PU) by using N30R83 atlas in PMOD; **(A)** shows the outlining of CN in sagittal, axial, and coronal planes; **(B)** shows the outlining of PU in sagittal, axial, and coronal planes.

Reconstructed images were transferred from the scanner workstation to a data analysis PMOD workstation (PMOD version 3.906 Software, Zurich, Switzerland) for biomedical image quantification in different VOIs in the brain. PMOD-PNEURO Brain VOIs based on the maximum probability atlas (Hammers-N30R83) (31) was used in segmenting brain regions. Figure 1 gives an analysis example. T1 weighted images were employed for outlining anatomical structures. The selected VOIs of the brain for this study are the CN and PU. Statistics associated with standard uptake value, such as maximum SUV (SUV_max_), mean SUV (SUV_mean_), and SD SUV (SUV_SD_), of each above VOIs with Cerebellar cortex (CC) were calculated in all the ^11^C-CFT and ^11^C-PiB brain images.

For the evaluation of image quality among segmented VOIs between reconstruction methods, two metrics were used on each VOI, that is, SNR and contrast. SNR of the segmented VOI was calculated as the difference between the VOI and background compared with the background noise shown in Equation 1.


(1) $$\begin{array}{l} SNR_{VOI}\;=\frac{Signal-Background\;}{\sigma_{B}\;}\; \end{array}$$

Where the signal is defined as the SUV_mean_ value in the segmented VOI, the background is defined as the SUV_mean_ value of the cerebellum cortex VOI and the σ_B_ (noise) in this formula is defined as the SUV_SD_ value of the background VOI. The use of cerebellum cortex VOI as the background is due to its homogeneous uptake patterns relative to other VOIs in the brain (17, 32–34).

In this work, contrast is defined as a ratio of signal to background.


(2) $$\begin{array}{l} Contrast_{VOI}=\frac{Signal}{Background\;} \end{array}$$

Further, to calculate the percentage difference, the TOF values were expressed as a percentage difference from non-TOF values (Equation 3), according to the previous literature (35).


(3) $$\begin{array}{l} \%RAD\left(SUV_{x}\right)=\frac{{\left(SUV_{x}\;TOF-\;SUV_{x}\;nonTOF\right)}^{*}100\%}{SUV_{x}\;nonTOF} \end{array}$$

### Statistical Analysis

The IBM SPSS version 23.0 software was used to compare the TOF *vs*. non-TOF measurements. The comparisons among the SUVs of different brain regions of TOF *vs*. non-TOF reconstruction methods, TOF-contrast *vs*. non-TOF-contrast, and TOF-SNR *vs*. non-TOF-SNR were analyzed using the paired *t*-test. Before the *t*-test, the data had been tested and the distribution was normality and variance was homogeneous. The value of *p* < 0.05 was considered statistically significant. Box-plots were generated to display the distribution of data.

## Results

### SUV_max_ and SUV_mean_

**Figure 2:**
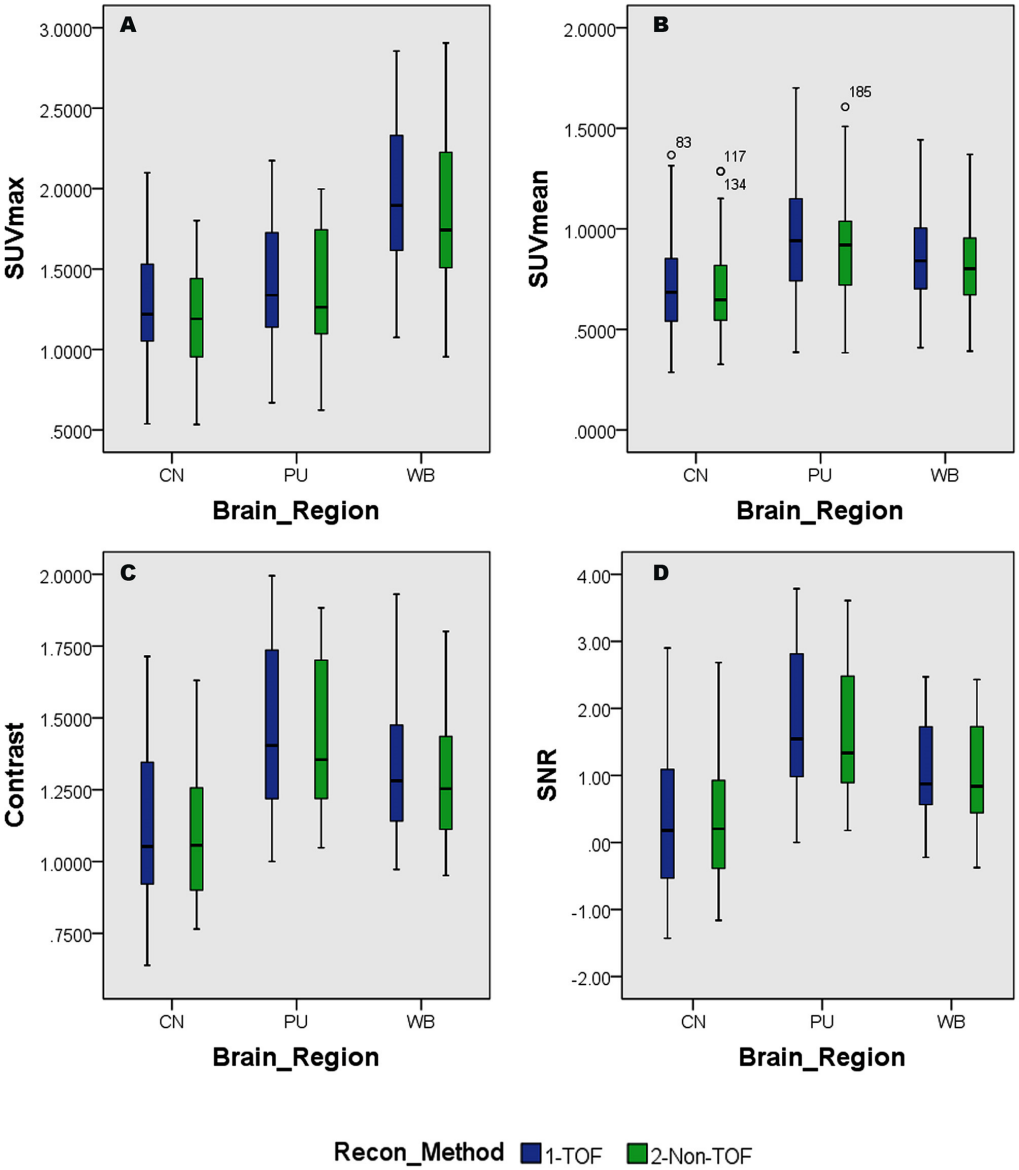
Distribution of the standard uptake values (SUVs) and quantitative parameters with reconstruction methods per each brain regions of ^11^C-PiB brain PET; **(A)** shows the distribution of individual subject's SUVmax values per segmented brain VOIs; **(B)** shows the distribution of individual subject's SUVmean values per segmented brain VOIs; **(C)** shows the distribution of individual subject's SNR values per segmented brain VOIs; **(D)** shows the distribution of individual subject's Contrast values per segmented brain VOIs; WB, Whole brain; CN, Caudate Nuclei; PU, Putamen.

**Figure 3:**
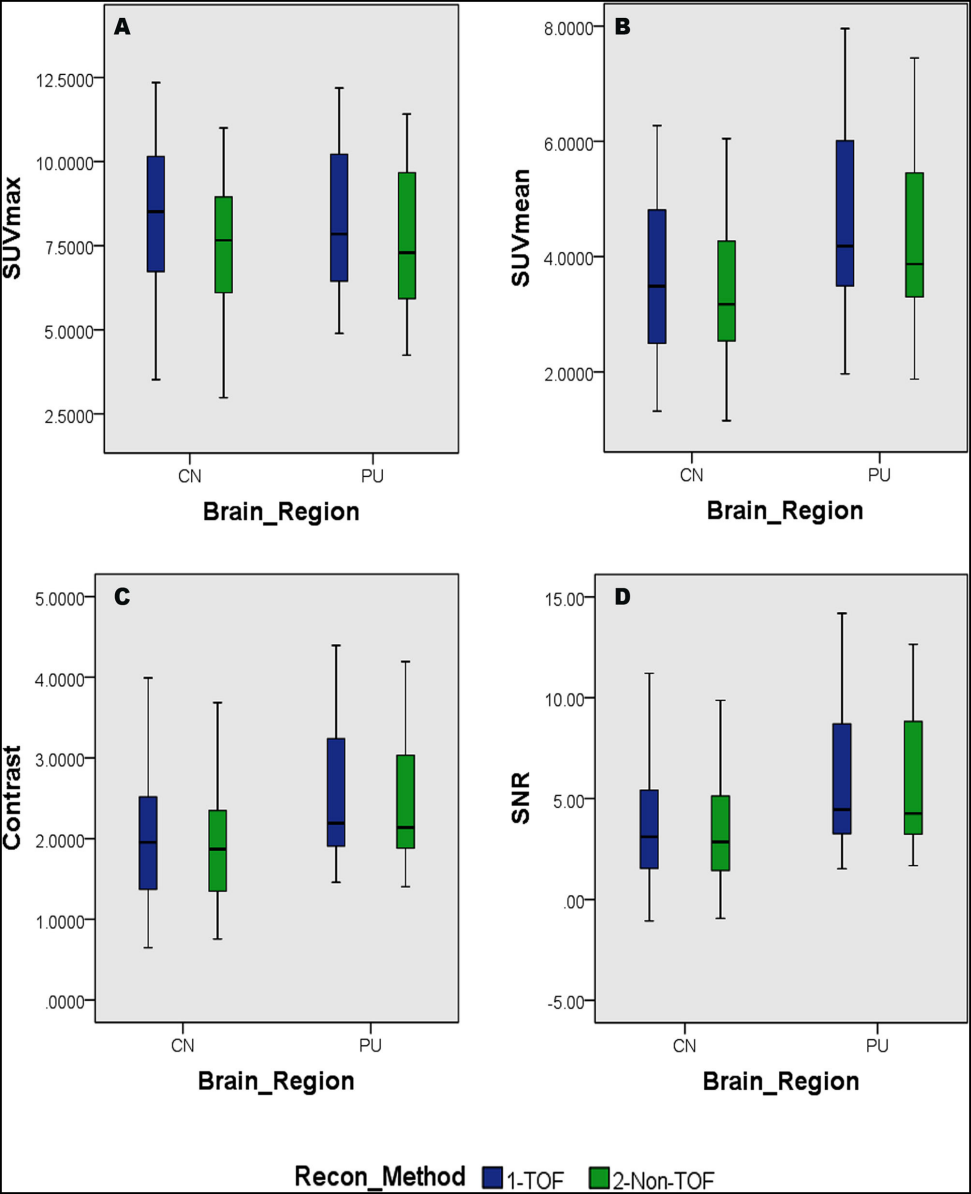
Distribution of the SUVs and quantitative parameters with reconstruction methods per each brain regions of ^11^C-PFT brain PET; **(A)** shows the distribution of individual subject's SUVmax values per segmented brain volume of interests (VOIs); **(B)** shows the distribution of individual subject's SUVmean values per segmented brain VOIs; **(C)** shows the distribution of individual subject's signal-to-noise ratio (SNR) values per segmented brain VOIs; **(D)** shows the distribution of individual subject's contrast values per segmented brain VOIs; WB, whole brain; CN, caudate Nuclei; PU, putamen.

Overall higher average SUV_max_ and SUV_mean_ values were observed among CN, PU regions, and whole brain in TOF compared with non-TOF reconstructions in ^11^C-CFT, and ^11^C-PiB brain images (Figures 2A,B, 3A,B). A statistically significant difference (*p* ≈ 0.000) was seen only in the CN region for SUV_max_ in TOF (1.293 ± 0.39) compared with non-TOF (1.192 ± 0.34) reconstruction in ^11^C-PiB, the similar impact was observed for whole-brain *p* ≈ 0.003 (1.966 ± 0.47, 1.869 ± 0.51). Statistically significant differences (*p* < 0.05) among both CN: *p* ≈ 0.000 (8.339 ± 2.31, 7.533 ± 2.16) and PU: *p* ≈ 0.004 (8.341 ± 2.28, 7.742 ± 2.13) regions for SUV_max_ in ^11^C-CFT were observed. Statistically significant differences (*p* < 0.05) were seen for all the VOIs segmented for SUV_mean_ in TOF compared with non-TOF reconstruction for both ^11^C-PiB and ^11^C-CFT (Table 2). Though few potential outliers were found for TOF and non-TOF reconstruction in both CN and PU regions for ^11^C-PiB (Figure 2B).

**Table 2:** The lists of maximum standard uptake value (SUVmax) and mean standard uptake value (SUVmean) for ^11^C-PiB and ^11^C-CFT with the time-of-flight (TOF) and non-TOF reconstruction, respectively.

**Type of radiotracer**	**Brain regions**	**Mean ±SD**							
		**SUVmax**				**SUVmean**			
		**TOF**	**Non-TOF**	**%RAD**	***p*-value**	**TOF**	**Non-TOF**	**%RAD**	***p*-value**
^11^C-PiB	WB	1.966 ± 0.47	1.869 ± 0.51	5.165	**0.003**	0.862 ± 0.24	0.822 ± 0.23	4.797	**5E-06**
	CN	1.293 ± 0.39	1.192 ± 0.34	8.513	**0.000**	0.730 ± 0.26	0.707 ± 0.23	3.334	**3E-02**
	PU	1.409 ± 0.39	1.366 ± 0.40	3.135	0.110	0.975 ± 0.32	0.925 ± 0.29	5.328	**2E-04**
^11^C-CFT	CN	8.339 ± 2.31	7.533 ± 2.16	10.702	**0.000**	3.613 ± 1.47	3.346 ± 1.29	7.981	**2E-04**
	PU	8.341 ± 2.28	7.742 ± 2.13	7.733	**0.004**	4.686 ± 1.60	4.301 ± 1.43	8.968	**2E-05**

*The mean ± SD represented the mean value and SD. WB, whole-brain; CN, caudate nuclei; PU, putamen; %RAD, percentage relative average difference. p < 0.05: difference is significant at the level of 0.05. The bold values are the significant ones*.

### SNR and Contrast

The SNR gain was measured in TOF and non-TOF by Equation 1. Overall all the VOIs with whole-brain showed higher SNR gain in TOF compared with non-TOF reconstruction in ^11^C-PiB (Figure 2D), significant SNR enhancement was observed in PU (*p* ≈ *0.034; 1.782* ± *1.08, 1.630* ± *0.99)* and whole-brain (*p* ≈ *0.018; 1.129* ± *0.73, 1.028* ± *0.71);* however, a significant difference in the result for the CN region is found. Similarly, for ^11^C-CFT, CN and PU, both regions showed higher SNR gain in TOF compared with non-TOF reconstruction (Figure 3D), yet significant improvement was not found in results as shown in Table 3.

**Table 3:** The quantitative parameters, such as the contrast and SNR were listed for the evaluation of image quality with TOF and non-TOF reconstruction, respectively.

**Type of radiotracer**	**Brain regions**	**Mean ±SD**							
		**Contrast**				**SNR**			
		**TOF**	**non-TOF**	**%RAD**	***p*-value**	**TOF**	**non-TOF**	**%RAD**	***p*-value**
^11^C-PiB	WB	1.311 ± 0.22	1.281 ± 0.20	2.347	**0.002**	1.129 ± 0.73	1.028 ± 0.71	9.901	**0.018**
	CN	1.103 ± 0.27	1.097 ± 0.23	0.507	0.690	0.382 ± 1.09	0.344 ± 0.95	10.936	0.525
	PU	1.469 ± 0.30	1.430 ± 0.27	2.693	**0.007**	1.782 ± 1.08	1.630 ± 0.99	9.326	**0.034**
^11^C-CFT	CN	1.964 ± 0.81	1.907 ± 0.73	2.997	0.138	3.496 ± 2.83	3.327 ± 2.57	5.069	0.174
	PU	2.527 ± 0.82	2.440 ± 0.75	3.571	**0.018**	5.767 ± 3.29	5.541 ± 3.08	4.068	0.076

*The mean ± SD represented the mean value and SD. WB: whole-brain; CN: caudate nuclei; PU: putamen; %RAD: percentage relative average difference. p < 0.05: Difference is significant at the level of 0.05. The bold values are the significant ones*.

Image contrast of all brain VOIs was measured in TOF and non-TOF by Equation 2. CN and PU regions and whole-brain showed average higher contrast in TOF compared with non-TOF for ^11^C-PiB (Figure 2C). Nevertheless, significant contrast (*p* < 0.05) improvement was observed only in PU (*p* ≈ *0.007; 1.469* ± *0.30, 1.430* ± *0.27)* region. However, the whole-brain showed a similar impact *(p* ≈ *0.002;* 1.311 ± 0.22, 1.281 ± 0.20*)* with significantly improved contrast in TOF reconstruction. In ^11^C-CFT, CN and PU showed higher contrast in TOF compared with non-TOF (Figure 3C), still significant contrast (*p* < 0.05) enhancement was observed only for PU region (*p* ≈ *0.018;* 2.527 ± 0.82, 2.440 ± 0.75*)* in TOF compared with non-TOF reconstruction.

### Percentage of Relative Average Difference of SUV_max_, SUV_mean_, and Quantitative Parameters—(% RAD)

The percentage of relative average difference (%RAD) of SUV_max_ and SUV_mean_ among segmented brain VOIs for both ^11^C-PiB and ^11^C-CFT was measured in TOF compared with non-TOF by Equation 3. The %RAD-SUV_max_ and SUV_mean_ difference for all segmented brain VOIs were positive, and %TOF SUV gain of ^11^C-CFT and ^11^C-PiB are illustrated in Table 2. The %RAD of SNR and contrast was positive for CN and PU regions for both ^11^C-CFT and ^11^C-PiB (Table 3).

## Discussion

**Figure 4:**
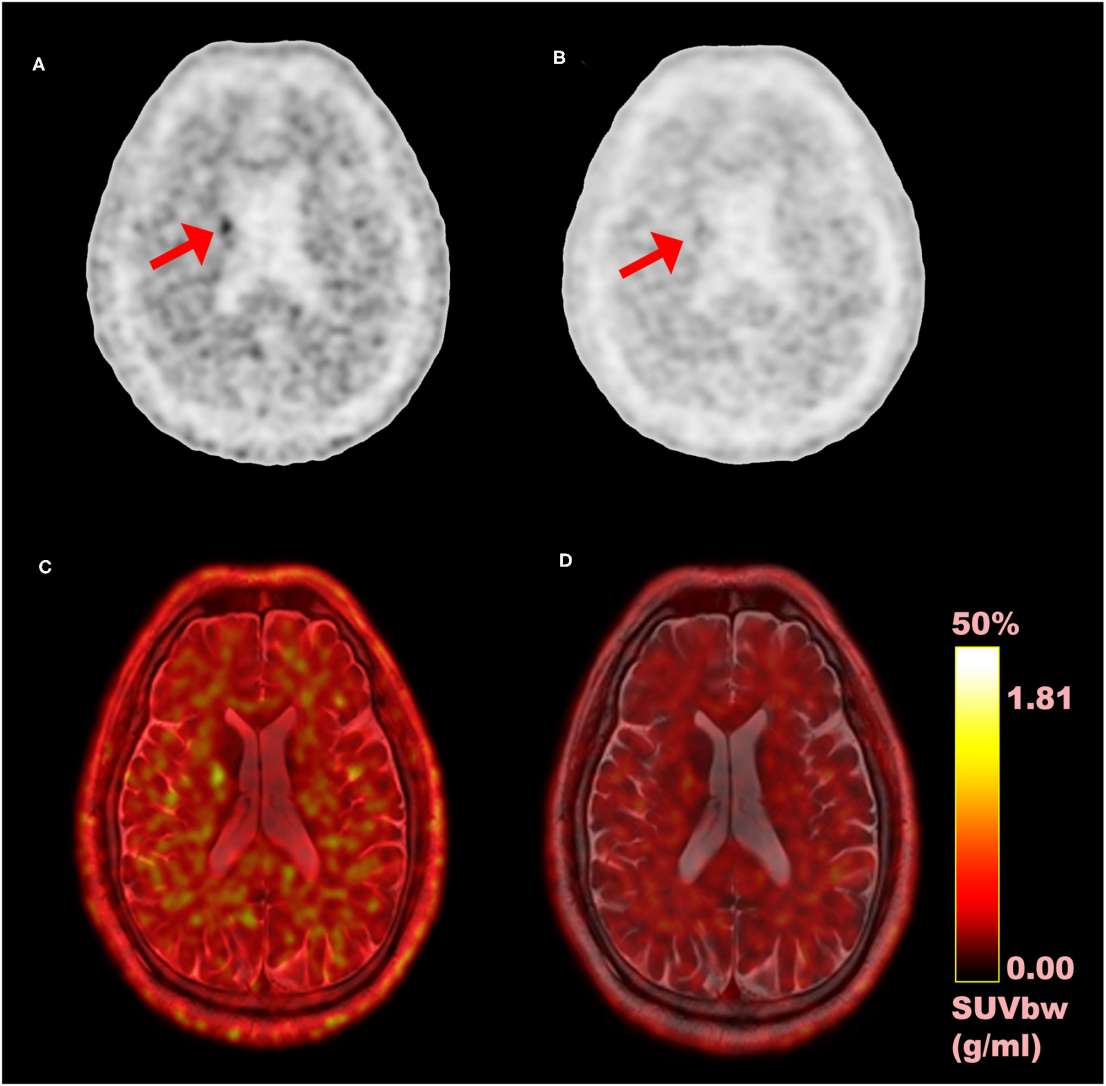
Comparison of time-of-flight (TOF) vs. non-TOF ^11^C-PiB PET images; **(A)** TOF reconstructed PET image (axial plane); **(B)** non-TOF reconstructed PET image (axial plane); **(C)** fusion image of TOF PET and MRI; **(D)** fusion image of non-TOF PET and MRI; red arrows: shows the signal enhancement difference in TOF and non-TOF PET images in ^11^C-PiB PET images.

In the study, we evaluated the magnitude of quantitative difference produced by TOF reconstructions on CN and PU VOIs for short-lived ^11^C-CFT brain PET and further compared with the same VOIs with ^11^C-PiB for any correlation of TOF effect with a different short-lived tracer. Apparently, varying uptake properties of different VOIs caused a considerable impact on the TOF effect; however, the TOF effect has a consistent association with SUV_mean_ rather than SUV_max_ values in both VOIs between ^11^C-CFT and ^11^C-PiB. Significantly enhanced SUV_mean_ among segmented VOIs of both radiotracers confirmed that TOF facilitates short-lived radiotracers over non-TOF reconstruction. Moreover, the whole-brain, which is investigated due to its diffuse ^11^C-PiB uptake qualities, spotted significantly enhanced SUV_max_, SUV_mean_, contrast, and SNR. It is observed that quantitative differences of image quality parameters vary among CN and PU with their uptake characteristics relative to the selected reference region. To the best of our knowledge, it is the first time that different tracers of short-lived ^11^C for brain quantitation imaging were performed with TOF and non-TOF PET/MRI. Overall, the experiment revealed that TOF reconstructions significantly affect SUVs compared with non-TOF and further improved the image contrast and SNR for a considerable extent, which proposed the TOF technique with higher time resolution (lesser than 400 ps) that contributes in achieving the optimal performance reconstruction of brain PET images with short-lived ^11^C-labeled tracers (Figure 4). Thus, it is recommended to consider the quantitative difference caused by TOF PET/MR modalities while diagnosing AD/PD.

The quantitative effect (SUVs) has benefitted in modern TOF PET for diagnosing neurodegenerative diseases by improving the spatial resolution and SNR (14). Further, Surti S et al. proved that the TOF reconstruction improves small lesion uptake measurement accuracy and precision by reducing normalized uptake values' (NUV) variability (22, 36). So as, the precision and accuracy of SUV are improved by TOF reconstruction. Oldan, J.D. et al. stated that SUV measurements of ^18^F-NaF PET/CT fluctuate within the brain like soft tissue regions between TOF and non-TOF reconstructions due to their lower uptake characteristics (35). Experimenting, the point spread function (PSF) and TOF algorithms on brain regions, Shao, X. et al., evidenced different effects on the SUVs among different brain regions for ^18^F-FDG (27). Since consistent significant enhancement of SUV_mean_ among segmented brain VOIs in TOF reconstruction for short-lived ^11^C-labeled tracers were seen, it is evidenced that TOF PET systems can be used as sensitivity amplifiers for short half-life radiopharmaceuticals, such as ^11^C-labeled tracers with low count rate after an adequate uptake time. Injected radiation dose can be optimized by considering enhanced SUVs while maintaining the same image quality. So the patient radiation dose, as well as occupational and general public exposure to ionizing radiation, can be minimized (23). Motion artifacts are often complained while scanning patients with AD/PD for longer time, however, improved timing resolution considerably reduce the scan time which comfort patients with less time inside the PET/MR gantry.

**Figure 5:**
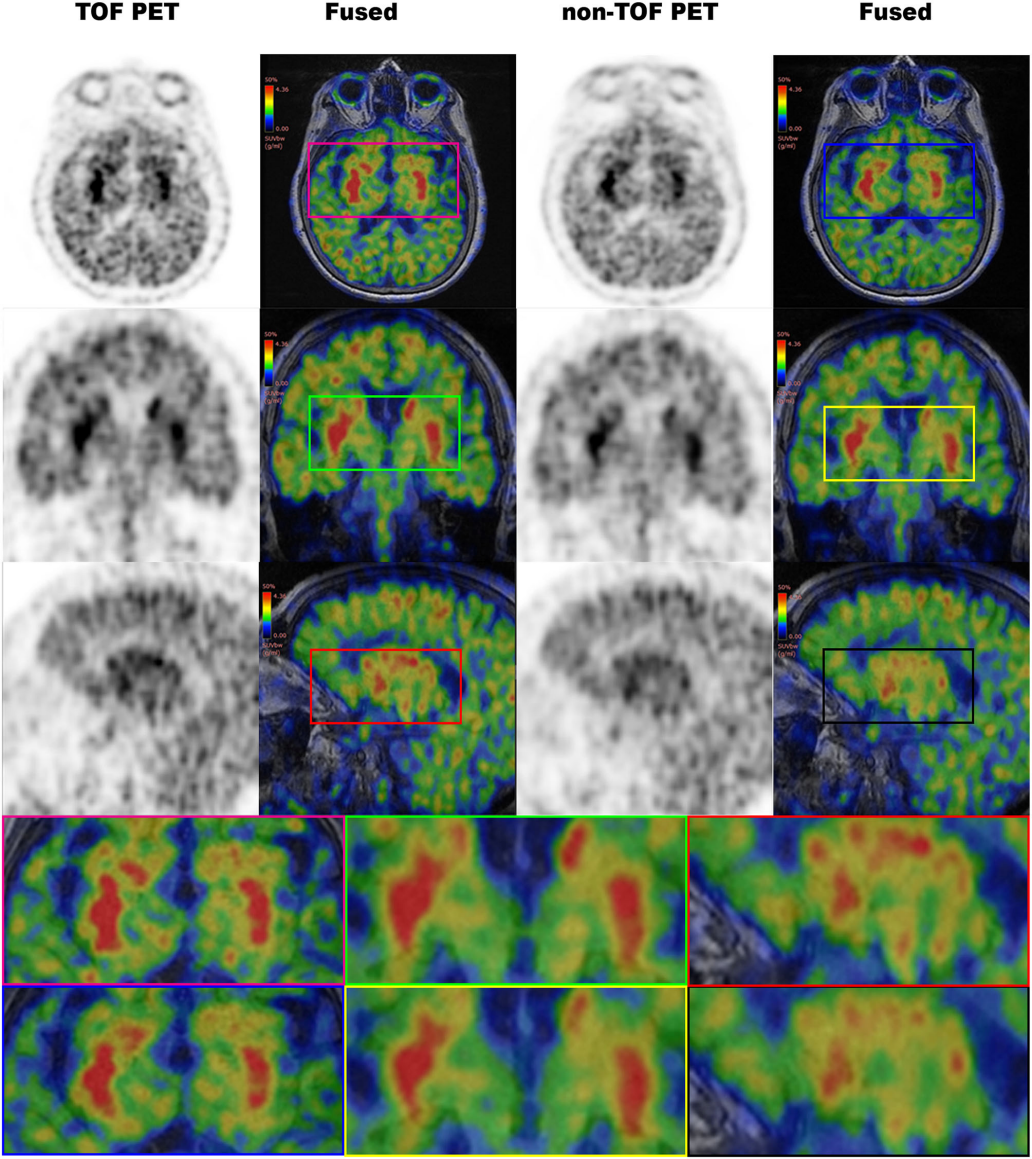
The ^11^C-CFT PET images demonstrating enhanced CN and PU with TOF and non-TOF reconstruction. First column demonstrating the enhanced CN and PU in TOF reconstruction; second column demonstrating the fused ^11^C-CFT with T1W MRI in TOF reconstruction; third column demonstrating the enhanced CN and PU in non-TOF reconstruction; fourth column demonstrating the fused ^11^C-CFT with T1W MRI in non-TOF reconstruction (axial, sagittal, and coronal planes are provided for comparisons).

**Figure 6:**
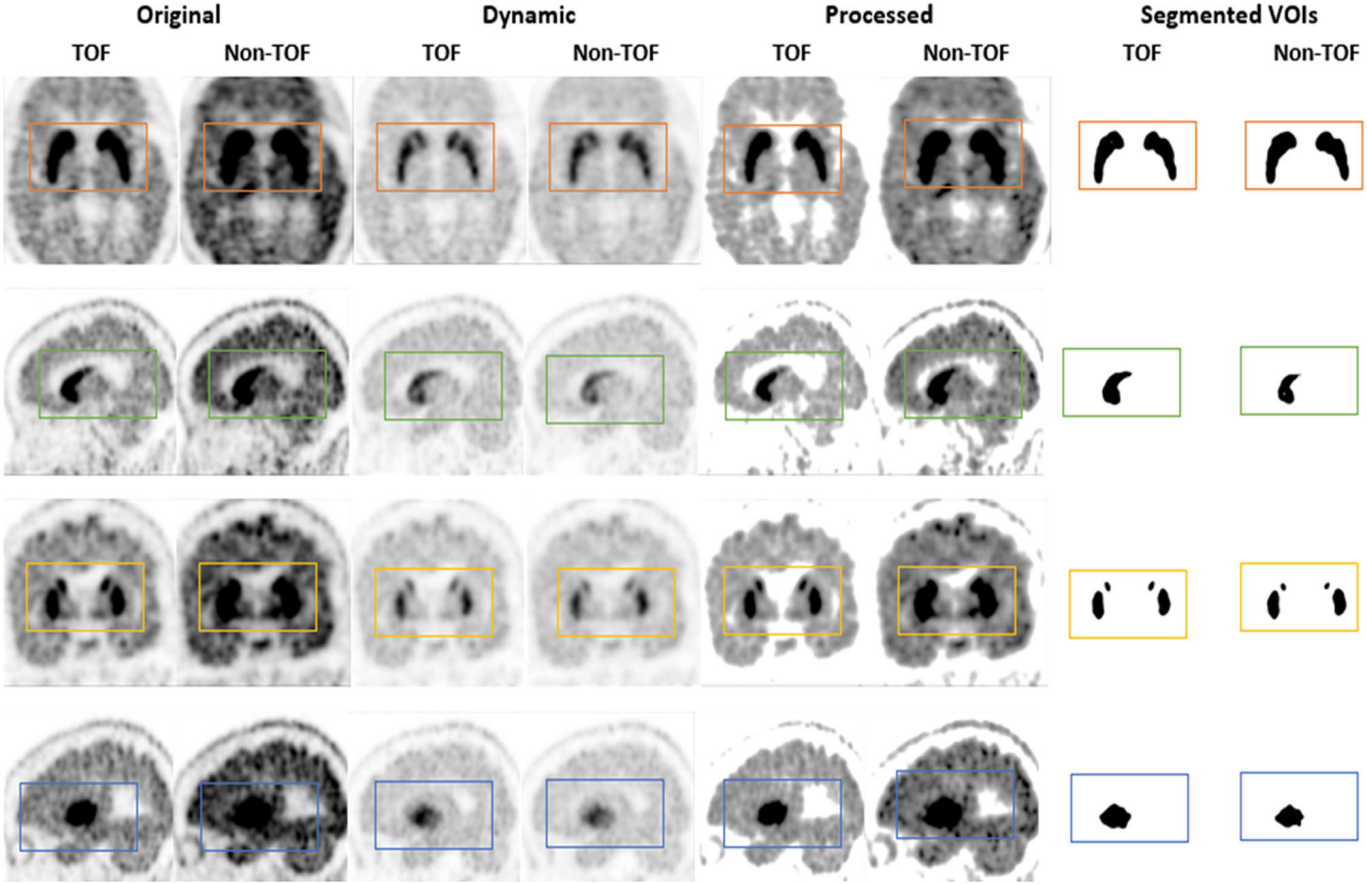
The ^11^C-CFT PET images demonstrating enhanced CN and PU with TOF and non-TOF reconstruction in original and processed images.

Caudate nuclei showed significant enhancement in SUV_max_ and SUV_mean_ for TOF reconstruction in ^11^C-CFT though a significant difference was not found in the results of the SNR and contrast. Similarly, ^11^C-PiB showed identical results for both the VOIs. These results were caused due to a relatively improved signal in the reference region, which is the cerebellum cortex, compared with the CN region in TOF reconstruction. PU region with ^11^C-PiB showed significant enhancement in contrast, SNR and SUV_mean_ for TOF compared with non-TOF reconstruction, which is consistent with previous literature using ^18^F tracers for small lesion enhancement (16, 18, 19). However, PU did not show significant improvement in SUV_max_ in TOF reconstructed images compared with non-TOF, which probably was due to diffuse low uptake properties of ^11^C-PiB tracer within the PU. A similar effect was observed in SUV_max_, SUV_mean_, and contrast for ^11^C-CFT due to its higher uptake characteristics within the PU, (Figures 5, 6) still SNR did not find significant development. The cause would be the incomparable noise produced in the background region, which is the cerebellum cortex (15), was relatively higher in TOF compared with non-TOF images due to ^11^C-CFT uptake properties. In Figure 2, the box-plot illustrated overall whole-brain enhanced SUV_mean_, SUV_max_, and quantitative parameters with TOF reconstruction in ^11^C-PiB.

Our study has several limitations. The number of cases are still limited due to the time limitation for data collection of ^11^C-CFT and ^11^C-PiB scans of suspected patients with AD/PD, which were archived in the Picture Archive and Communication System (PACS). A larger sample would possibly be needed to generalize these findings to a considerable population (e.g., a wider range of patient BMI and a wider range of age). Then, for outlining most of the cortical structures in the brain VOIs, PET-based Maximum Probability Atlas (MPA) was used to avoid slowness or interruption of the segmentation process of the PNEURO module in PMOD 3.9 software. Though T1 MR based parcellation is preferred over VOI outlining in deep nuclei region by the PMOD team, thus the quality of the VOI definition in the areas mentioned above is reduced. For the effective use of PNEURO with high-resolution data, a high-end workstation (e.g., 8 core, 16GB, or more RAM) is required.

## Conclusion

Time-of-flight reconstruction improves SUVs and image quality parameters, which is an advantage of the TOF PET/MRI system with short-lived ^11^C-labeled tracers for offering higher sensitivity. The improved temporal resolution supports the rapid decay rate of short-lived ^11^C-labeled tracers and shortens scan time while increasing the patient comfort and reducing the motion artifacts in patients with AD/PD. However, the combined TOF algorithm should be used with caution for quantitative analysis because it has different effects on SUV_max_, contrast, and SNR of different brain regions.

## Data Availability Statement

The original contributions presented in the study are included in the article/supplementary material, further inquiries can be directed to the corresponding author.

## Ethics Statement

The studies involving human participants were reviewed and approved by Institutional Review Board of Union Hospital, Tongji Medical College, Huazhong University of Science and Technology. Written informed consent for participation was not required for this study in accordance with the national legislation and the institutional requirements.

## Author Contributions

XL and WR substantially contributed to the conception and design, analyzed and interpreted the data, and revised the manuscript critically for important intellectual content. DW analyzed and interpreted the data and drafted the article. DW, WR, and FH acquired the PET images. YG and QL prepared the compounds of ^11^C-CFT and ^11^C-PiB. XS and FL analyzed and interpreted the images. All authors read and approved the final manuscript, contributed to the article, and approved the submitted version.

## Funding

This work was supported by the National Natural Science Foundation of China (Nos. 81701759 and 81901735), the Key Project of Hubei Province Technical Innovation (2017ACA182), and the Clinical Research Physician Program of Tongji Medical College, Huazhong University of Science and Technology (No. 5001530008).

## Conflict of Interest

The authors declare that the research was conducted in the absence of any commercial or financial relationships that could be construed as a potential conflict of interest.

## Publisher's Note

All claims expressed in this article are solely those of the authors and do not necessarily represent those of their affiliated organizations, or those of the publisher, the editors and the reviewers. Any product that may be evaluated in this article, or claim that may be made by its manufacturer, is not guaranteed or endorsed by the publisher.
